# Efficacy of pyrazinoic acid dry powder aerosols in resolving necrotic and non-necrotic granulomas in a guinea pig model of tuberculosis

**DOI:** 10.1371/journal.pone.0204495

**Published:** 2018-09-27

**Authors:** Stephanie A. Montgomery, Ellen F. Young, Phillip G. Durham, Katelyn E. Zulauf, Laura Rank, Brittany K. Miller, Jennifer D. Hayden, Feng-Chang Lin, John T. Welch, Anthony J. Hickey, Miriam Braunstein

**Affiliations:** 1 Department of Pathology and Laboratory Medicine and Lineberger Comprehensive Cancer Center, School of Medicine, University of North Carolina, Chapel Hill, North Carolina, United States of America; 2 Department of Microbiology and Immunology, School of Medicine, University of North Carolina, Chapel Hill, North Carolina, United States of America; 3 RTI International, Research Triangle Park, North Carolina, United States of America; 4 Department of Biostatistics and North Carolina Translational and Clinical Sciences Institute, University of North Carolina, Chapel Hill, North Carolina, United States of America; 5 Department of Chemistry, University at Albany, Albany, New York, United States of America; Public Health England, UNITED KINGDOM

## Abstract

New therapeutic strategies are needed to treat drug resistant tuberculosis (TB) and to improve treatment for drug sensitive TB. Pyrazinamide (PZA) is a critical component of current first-line TB therapy. However, the rise in PZA-resistant TB cases jeopardizes the future utility of PZA. To address this problem, we used the guinea pig model of TB and tested the efficacy of an inhaled dry powder combination, referred to as Pyrazinoic acid/ester Dry Powder (PDP), which is comprised of pyrazinoic acid (POA), the active moiety of PZA, and pyrazinoic acid ester (PAE), which is a PZA analog. Both POA and PAE have the advantage of being able to act on PZA-resistant *Mycobacterium tuberculosis*. When used in combination with oral rifampicin (R), inhaled PDP had striking effects on tissue pathology. Effects were observed in lungs, the site of delivery, but also in the spleen and liver indicating both local and systemic effects of inhaled PDP. Tissue granulomas that harbor *M*. *tuberculosis* in a persistent state are a hallmark of TB and they pose a challenge for therapy. Compared to other treatments, which preferentially cleared non-necrotic granulomas, R+PDP reduced necrotic granulomas more effectively. The increased ability of R+PDP to act on more recalcitrant necrotic granulomas suggests a novel mechanism of action. The results presented in this report reveal the potential for developing therapies involving POA that are optimized to target necrotic as well as non-necrotic granulomas as a means of achieving more complete sterilization of *M*. *tuberculosis* bacilli and preventing disease relapse when therapy ends.

## Introduction

The global health crisis posed by tuberculosis (TB) is immense. In 2016 alone, 10.4 million individuals became sick with *Mycobacterium tuberculosis* (*M*. *tuberculosis*), the infectious bacterium that causes TB, and 1.7 million people died from TB [1]. Moreover, ongoing efforts to control TB are increasingly challenged by multidrug resistant (MDR) and extensively drug resistant (XDR) *M*. *tuberculosis* strains.

Despite commendable efforts, new TB drug and vaccine development is progressing slowly. In the last thirty years, only two new drugs, bedaquiline and delaminid, were introduced to treat TB. These new drugs alone will not solve the TB problem [2]. Moreover, there are no new vaccines to replace the live attenuated *M*. *bovis* BCG (Bacille Calmette Guerin) vaccine that was introduced almost a century ago and does not reliably protect against adult TB [3]. To solve the global TB crisis, a robust pipeline of new therapies is needed. As an alternative to developing entirely new drugs with novel mechanisms of action, a faster path to new therapies may be to revisit and optimize already approved TB drugs [4,5]. Such optimization could entail rescuing activity that is lost on drug resistant *M*. *tuberculosis* or improving activity on drug sensitive *M*. *tuberculosis*. Another possibility is that direct delivery of drugs to the lungs, the primary site of *M*. *tuberculosis* infection, could enhance the efficacy of TB therapies.

Inhaled therapy for TB was first considered almost 70 years ago. In the late 1940s, streptomycin aerosols were administered on a case-by-case basis to treat serious life-threatening *M*. *tuberculosis* infections [6,7]. Although there was early clinical success with this off-label strategy, introduction of the orally delivered TB drugs that are still in use today (rifampicin, isoniazid, ethambutol and pyrazinamide) proved to be quite effective and further development of inhaled TB therapies halted at that time.

Given the desperate need for new and improved TB treatments, particularly with the rise in MDR and XDR TB, there is interest in resurrecting inhaled therapies for TB [8,9]. Inhaled delivery has potential to improve drug access to bacteria in the lung and, more specifically, to *M*. *tuberculosis* contained in pulmonary granulomas. Inhaled therapies could be used to supplement TB drugs delivered by conventional routes as an approach for shortening the lengthy duration of current treatment (6 months for drugs sensitive TB and ≥18 months for drug resistant TB) [1] and/or to limit the transmission period by more rapidly clearing the airway. Further, by maximizing the drug concentration in the lung, inhaled TB therapy could reduce drug dosage and systemic side effects. Studies of inhaled TB drugs on *M*. *tuberculosis* infected animals demonstrate the feasibility of this approach [8,10–19].

Although there are a few examples of inhaled TB drugs reaching clinical assessment [20], current research on inhaled TB therapy is largely in preclinical development [21]. However, for other pulmonary diseases, such as asthma and chronic obstructive pulmonary disease, aerosol therapy is well established and in clinical use [8]. Three major categories of inhaled drug delivery systems exist: pressurized metered dose inhalers, dry powder inhalers (DPI) and nebulizers. Since DPI products are portable and avoid the need for special packaging, needles, cold storage, or electricity they are well suited for application in TB high-burden countries. An approach for developing DPI products has been described [8].

In this study, we investigated the potential of treating TB with inhaled pyrazinamide analogs. Pyrazinamide (PZA) is one of the four drugs that comprise first-line combination therapy for TB, and it is considered to be an irreplaceable component of the regimen due to its effect of shortening therapy from nine to six months [22]. In a granuloma, *M*. *tuberculosis* is in a state of non/slow-replicating persistence [23,24], and PZA is particularly effective on non-replicating *M*. *tuberculosis* [22]. Additionally, PZA is able to penetrate granulomas [25]. In TB patients receiving treatment, PZA delivered by conventional oral dosing reaches the necrotic acellular core as well as the surrounding cellular regions of granulomas, which is not the case for all TB drugs [26]. Further, PZA acts in hypoxic, acidic, and nutrient limiting conditions, which may exist in granulomas, regions of inflammation, or the intracellular environment encountered by *M*. *tuberculosis* during infection [27–31].

When combined with other drugs [32,33], PZA has sterilizing and synergistic effects on *M*. *tuberculosis*. Consequently, PZA is often included when treating MDR-TB [22] or testing new TB drug candidates [34]. However, the future utility of PZA is threatened by the emergence of PZA-resistance (~50% of MDR *M*. *tuberculosis* are PZA-resistant) [35–37]. PZA is a prodrug that is converted to the active moiety pyrazinoic acid (POA) by the PncA pyrazinamidase of *M*. *tuberculosis* [38]. PncA-deficient mutants of *M*. *tuberculosis* are unable to convert PZA to POA and they account for the majority of PZA-resistant strains (78–85% in various studies) [39–43]. Consequently, PZA analogs such as pyrazinoic acid esters (PAEs) that undergo hydrolysis to form POA without the need for PncA or POA itself circumvent *pncA* deficiency. As a result, PAEs and POA are effective on the largest category of PZA resistant strains, and they present potential strategies to rescue the use of PZA for drug resistant disease [44–50].

We recently tested the efficacy of PAE aerosols, delivered as an aqueous dispersion from a nebulizer, combined with oral rifampicin (R) in treating *M*. *tuberculosis* infected guinea pigs and observed a reduction in bacterial burden compared to untreated animals [15]. Motivated by these past results and the advantageous properties of DPIs, here we tested efficacy of a dry powder combination of POA and a PAE in treating TB in guinea pigs. Our results reveal this Pyrazinoic acid/ester Dry Powder (PDP) to be effective as an adjunctive antitubercular chemotherapy. Used in combination with oral rifampicin (R), inhaled PDP had both local (lung) and extrapulmonary systemic (spleen and liver) effects. Most striking was our discovery that, while all treatments tested reduced the number of pulmonary granulomas, the R+PDP treatment was better able to reduce the percent of lung tissue affected by necrosis and resolve necrotic granulomas than other treatments.

## Materials and methods

### Ethics statement

All experimental protocols were approved with written consent by the Institutional Animal Care and Use Committee of the University of North Carolina (IACUC # 15–125). Guinea pigs were euthanized by injection with sodium pentobarbital (Socumb) from Henry Schein Animal Health (Columbus. OH).

### Reagents

n-Propyl pyrazinoate (PAE) used in this study was prepared by previously established methods and confirmed by NMR [51]. Maltodextrin, POA and reagent grade ethanol were obtained from Sigma-Aldrich (St. Louis, MO). L-leucine was obtained from Alfa Aesar (Ward Hill, MA). PZA was obtained from ACROS Organics(Morris Plains, NJ).

### Formulation

#### Pyrazinoic acid/ester dry powder (PDP)

A spray dried aerosol powder was prepared by combining POA and PAE using a method described previously [52]. Briefly, maltodextrin, leucine, POA and PAE were combined into an aqueous solution and spray dried (Buchi B-290, Flawil, Switzerland). Resulting particles were characterized to determine aerodynamic particle size distribution (NGI, MSI Corp, USA) and POA/PAE content (Acquity UPLC, Waters, USA).

#### Nebulized PAE

For the nebulized PAE comparison group, a 15% PAE solution in 25% ethanol/water was nebulized from an Aeroneb 4–6 μm volume median diameter nebulizer head and Aeroneb Lab control module (Aerogen, Dangan Galway, Ireland).

### Guinea pig treatment efficacy studies

#### Animals

All animal experiments were performed in an animal Biosafety Level 3 facility at the University of North Carolina Chapel Hill (UNC-CH). Male Hartley guinea pigs (250-300g) were purchased from Hilltop Labs (Scottsdale, PA). Guinea pigs were housed singly with a plastic dumbbell as enrichment and provided food and water ad libitum. Animals were allowed to acclimate 1 week prior to infection with *M*. *tuberculosis*. Animal health was monitored daily and body weights of each animal were recorded throughout the study. Criteria for euthanasia due to morbidity included unresponsiveness, labored breathing, cachexia. No animals exhibited outward signs of disease or lost weight during the course of this study.

#### *Mycobacterium tuberculosis* infection

For aerosol infection of guinea pigs with *M*. *tuberculosis*, the virulent H37Rv strain (William Jacobs Jr., Albert Einstein College of Medicine, Bronx NY) was first grown in liquid Middlebrook 7H9 medium (Becton Dickenson–BD) supplemented with 0.05% Tween 80, 0.5% glycerol,10% Middlebrook OADC Enrichment (BD) until mid-log phase (optical density at 600nm of 0.5–1.0). The bacterial culture was then washed and resuspended in PBS with 0.05% Tween 80 (PBS-Tw). The optical density of the washed culture was determined and, using the conversion of an OD_600_ of 1.0 being 2.5X10^8^ CFU/ml (determined previously by plating cultures), the culture was then diluted to a concentration of 3X10^5^ CFU/ml and placed in the nebulizer jar of a whole-body exposure aerosol chamber (Mechanical Engineering Workshop, Madison, WI). Guinea pigs placed in the chamber were exposed to H37Rv aerosols for 5 min. followed by a 20 min. purge of the chamber. This dosing yields ∼40 viable bacilli per guinea pig lung as determined by plating the lungs of infected animals in their entirety one day post-infection in an independent experiment. *M*. *tuberculosis* infection was allowed to develop for four weeks, which is a period of time sufficient for extrapulmonary dissemination to occur, for the bacterial burden in the lungs and spleen to plateau, and for granuloma formation to occur [53–57]. At four weeks post-exposure, drug treatment was initiated.

#### Aerosol drug treatment

Groups of six guinea pigs were placed in a custom-built nose only exposure chamber [15,52]. The same exposure chamber was used to disperse the PDP (PAE and POA combination dry powder) or PAE nebulized formulations, each in five equal doses, totaling 100mg, spread out over 20 min. The standing cloud of dispersed powder or liquid droplets was allowed to clear after each of the five boluses. Inhaled drug dose was estimated (as shown in S1 File) using parameters of mass of drug in particles, exposure conditions (mass of particles suspended in volume of air) and guinea pig respiration (breathing frequency and tidal volume). It should be noted, that due to biological variation in physiology and pulmonary function, dose ranges around the values calculated would be anticipated experimentally. Based on these calculations, approximately 5.8 and 0.3 mg/Kg of PAE, which has the potential to be converted by esterases into 4.3 and 0.2 mg/Kg of the active moiety POA, were delivered as a nebulized solution or dry powder, respectively, and 1.2 mg/Kg of POA alone was additionally present in the PDP. Thus, the aggregated potential dose of POA in the PDP powder was 1.4 (1.2 + 0.2) mg/Kg versus 4.3 mg/Kg in the nebulized solution. Since the PDP also includes additives the total daily dose of PDP was calculated to be 3.9 mg/Kg, which is similar to doses used previously in other studies of inhaled TB drugs in guinea pigs [16].

This inhaled PDP dose equates to a human dose [58], based on average body weight (70Kg), of 273mg of PDP containing the aggregated potential dose of 100mg of POA. This dose is comparable to the 112mg dose of dry powder tobramycin currently used to treat *Pseudomonas aeruginosa* in Cystic Fibrosis patients [59]. The nebulized PAE dose used was equivalent to the one used previously [15].

#### Drug treatment protocols

**Fig 1:**
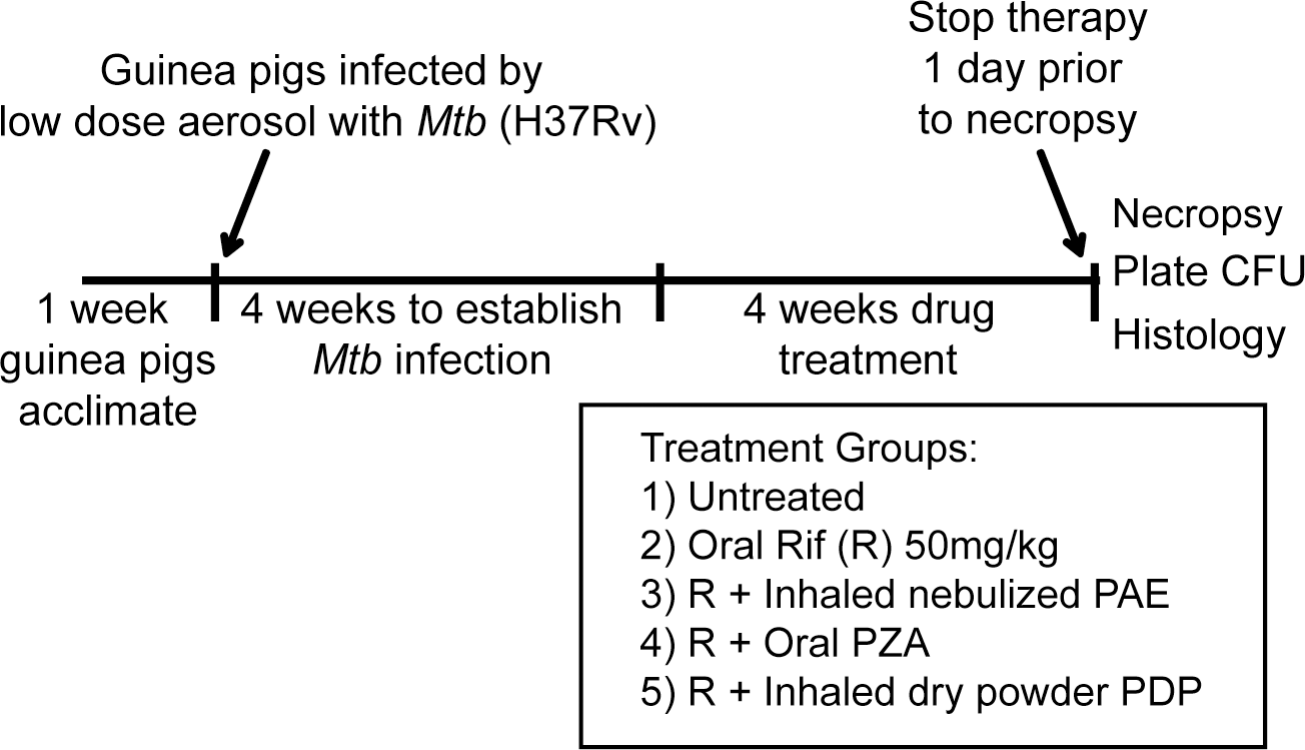
Timeline for drug efficacy evaluation using the guinea pig model of TB.

There were five treatment groups as follows: rifampicin (R), R plus inhaled nebulized PAE (R+PAE), R plus oral PZA (R+PZA), R plus inhaled dry powder PDP (R+PDP), and untreated (Un) (Fig 1). Treatments were given six days/week. Rifampicin (R) 50mg/Kg was made up in an oral delivery solution of 40% sucrose (w/v), 20%pumpkin puree (w/v) solution containing 50mg/Kg vitamin C and 1% *Lactobacillus acidophilus* and *Lactobacillus bulgaricus* (Lactinex, BD) as described [60]. Each day, R dosing preceded PDP, PAE or PZA dosing by at least 30 min. to limit possible drug antagonism [61]. Guinea pigs were dosed with aerosolized PDP or PAE by nose only inhalation in a custom-built chamber [52]. PZA was prepared as a liquid formulation in the same oral delivery solution described above for R dosing and was delivered orally at 25mg/Kg. The untreated group received the same oral delivery solution minus any drug. All oral dosing was achieved by passive feeding of 200μl of solutions dispensed from a syringe. Treatment commenced four weeks post-infection and lasted for an additional four weeks.

#### Bacterial burden determination at the end of the study

After four weeks of treatment, animals were removed from therapy for one day and then euthanized and necropsy performed. Guinea pigs were euthanized by injection with sodium pentobarbital (Socumb) from Henry Schein Animal Health (Columbus. OH).

The treatment-free day was included to allow time for any residual drug to be metabolized to avoid drug carryover when plating bacteria from organ homogenates. Spleens, lungs, and tracheobronchial lymph nodes were removed and a portion of each organ was weighed, and homogenized with a Seward Stomacher apparatus (Worthing, UK) in 5 ml PBS-Tw with 100μg/ml cyclohexamide and 50μg/ml carbenicillin. Organ homogenate was serially diluted and 100μl volumes of undiluted and diluted homogenates were plated in duplicate on 7H11 Middlebrook agar (BD) supplemented with 0.5% glycerol, 10% Middlebrook OADC Enrichment (BD), 100μg/ml cyclohexamide, 50μg/ml carbenicillin and 15μg/ml trimethoprim all from Sigma-Aldrich (St. Louis, MO). Colony forming units (CFU) were counted after three weeks incubation at 37°C and CFU/gram (g) tissue determined. The limit of detection was 10 CFU/g.

#### Histopathology

At necropsy, portions of three lung lobes (left and right caudal lobes and the left cranial lobe), tracheobronchial lymph nodes, trachea, two liver lobes, and spleen were removed and fixed by submersion in 10% neutral buffered formalin. Specimens were processed, embedded in paraffin wax, sectioned at four microns-thick, and haemotoxylin and eosin (H&E) stained. In order to analyze 2cm^2^ of each tissue from each guinea pig, between two to four sections of lung, two sections of spleen, two sections of liver, and two to three sections of lymph nodes were evaluated in a blinded fashion for histopathologic changes by a board-certified anatomic veterinary pathologist (S.A.M). Tissues from an uninfected animal were included for comparison. The percent tissue affected by granulomas was quantified, as described below. In addition, lung granulomas were counted and identified as necrotic or non-necrotic. For extrapulmonary tissues, the degree of pathology was scored as minimal, mild, moderate or severe according to parameters that included lesion distribution and size, types of macrophages present, and arrangement of lymphocytes (see S1 Table for descriptions and scoring parameters).

#### Slide digitalization and quantification of percent lung or spleen tissue affected by granulomas or percent of lung tissue affected by necrosis

H&E slides were scanned on an Aperio ScanScope XT (Leica Biosystems) using a 20X power objective. Images were acquired as JPEG2000-compressed Aperio SVS files at 8-bit depth with a resolution of 0.4942 microns per pixel. Compression quality was set to 70. Digital slides were viewed in ImageScope v12.3 (Leica Biosystems). Using Aperio ImageScope software, granulomas or regions of nerosis on each slide were demarcated, the area was measured and the percent total tissue on a slide affected quantitated.

#### Statistical methods

All data in this study were tested for normal distribution using D’Agostino & Pearson’s test and were analyzed accordingly. For the bacterial burden experiments, the data (CFU/g of tissue) were determined to be non-normally distributed and group means were compared using the non-parametric Kruskal-Wallis test. The data were log-transformed for normalization and stable variation. Means and standard errors were reported. Multiple comparisons between groups were adjusted by Dunn’s test (for non-normality) with p<0.01. The data for percent of lung or spleen tissue affected by granulomas and for percent of lung tissue affected by necrosis were determined to be non-normally distributed. For these data, we compared individual groups to the untreated group (Tables 1 and 4) or to the R group (Table 2) using the Mann-Whitney test with p<0.01. The data for percent of granulomas that were necrotic were determined to be normally distributed. For this data we compared individual groups to the R group using two-sample Student’s t-tests with p<0.05.

**Table 1:** Mean percent lung tissue affected by granulomas.

	4 week infection plus 4 week treatment				
	Untreated	R	R+PAE	R+PZA	R+PDP
Percent lung tissue affected ± SEM	23.4 ± 8.0	14.4 ± 3. 6	7.5 ± 1.2	7.3 ± 1.9	7.1 ± 1.5

**Table 2:** Mean percent lung tissue affected by necrosis.

	4 week infection plus 4 week treatment				
	Untreated	R	R+PAE	R+PZA	R+PDP
Percent lung tissue affected ± SEM	0.52 ± 0.25	0.68 ± 0.22	0.26 ± 0.12	0.18 ± 0.08	0.01 ± 0.01

**Table 3:** Assessment of histologic inflammation in extrapulmonary organs*.

Organ	4 week infection plus 4 week treatment				
	Untreated	R	R+PAE	R+PZA	R+PDP
Spleen	severe	moderate	moderate	moderate	minimal
Liver	severe	mild	mild	none	none
Tracheobronchial Lymph Node	severe	severe	severe	severe	severe

*Descriptive terms are defined in S1 Table.

**Table 4:** Mean percent spleen tissue affected by granulomas.

	4 week infection plus 4 week treatment				
	Untreated	R	R+PAE	R+PZA	R+PDP
Percent spleen tissue affected ± SEM	34.7 ± 6.1	2.6 ± 1.6	1.8 ± 1.3	2.3 ± 1.5	0.1 ± 0.1

## Results

### Pyrazinoic acid/ester dry powder (PDP) formulation

We set out to test the efficacy of inhaled dry powders of n-propyl pyrazinoate, hereafter referred to as PAE, with the goal of extending our past studies of nebulized pyrazinoic acid esters as an adjunctive therapy for treating TB[15]. PAE is a liquid at room temperature. Therefore, to prepare PAE dry powders we combined PAE with POA, as the latter is a solid. In this formulation, PAE could be trapped in the solid crystalline structure of POA, which is the biologically active product of PAE hydrolysis. Notably, the final composition of the pyrazinoic acid/ester dry powder (referred to as PDP) has a larger mass proportion of POA than PAE: 30.5% POA, 8.5% PAE, 30.5% maltodextrin and 30.5% leucine (maltodextrin and leucine serve as excipients) [52]. Dry powder particles were prepared by spray drying and physiochemical characterization performed. Aerodynamic particle size distribution (APSD) was determined by inertial impaction with the mass median aerodynamic diameter (MMAD) for PDP being 2.8 and a geometric standard deviation (GSD) of 1.7 [52]. The APSD of the nebulized liquid PAE solution that was tested alongside PDP in subsequent experiments had a MMAD of 2.7 and GSD of 1.6 [15]. These size ranges of PDP and PAE particles are suitable for aerosol delivery.

### PDP effects on bacterial burden in *Mtb* infected guinea pigs.

Guinea pigs were infected by the aerosol route with a low dose of virulent H37Rv *M*. *tuberculosis*. Following a four week period to establish infection, which is sufficient time for the bacterial burden in the lungs to plateau and for *M*. *tuberculosis* dissemination to the spleen to occur [53–57], a subsequent four week period of drug dosing six days a week was carried out (Fig 1).

**Fig 2:**
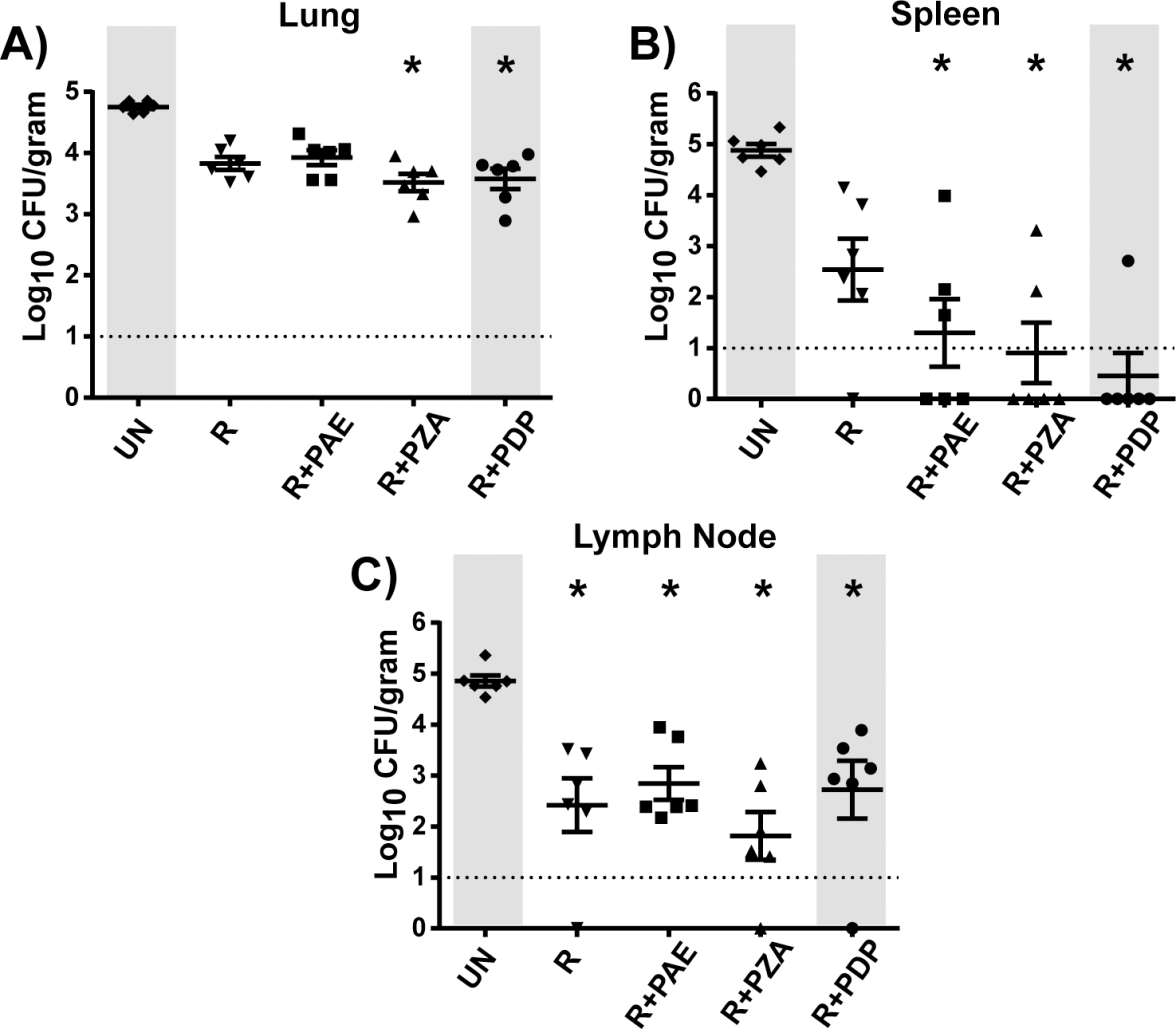
**Bacterial burden in lungs (A), spleen (B), or tracheobronchial lymph node (C).** Groups of six guinea pigs were infected with *M*. *tuberculosis* for four weeks and then treated six days a week for four weeks with oral rifampicin (R) alone or in combination with inhaled nebulized PAE, oral PZA or inhaled PDP. Following necropsy, bacterial burden was determined by plating organ homogenates for viable bacteria (colony forming units, CFU). The data are reported as mean Log_10_ CFU/gram organ weight. Error bars represent standard error of the mean. There were no significant differences between the R group and any other group. However, when compared to untreated (UN) animals using the non-parametric Kruskal-Wallis test followed by a Dunn’s test to calculate multiplicity adjusted p values (*p<0.01), some treatments exhibited significant differences in CFU. The level of detection is indicated in the graph as a dotted line. Animals with no detectable CFU were counted as having CFU at the level of detection.

Groups of animals were treated with oral rifampicin (R) alone or with inhaled PDP dry powder (R+PDP) or inhaled PAE (R+PAE), the latter being delivered as liquid aerosols by nebulization. An additional group of animals was treated with oral R plus oral PZA (R+PZA). Finally, an untreated group of animals that received no drugs was included as a control (Untreated). Compared to conventional oral doses, the estimated dose of the inhaled treatments was relatively low. The daily calculated dose of PDP was 3.9 mg/Kg, which included leucine and maltodextrin additives. This amount of PDP could provide an estimated 1.4 mg/Kg of POA, which includes 0.2 mg/Kg POA potentially released from PAE by esterases. The estimated daily dose of the nebulized liquid PAE solution was 5.8 mg/Kg (delivering a higher potential dose of 4.3 mg/Kg POA) (see S1 File for dose estimation calculations). Oral PZA was provided at a higher dose of 25mg/Kg. No adverse effects were observed with any treatment, including inhaled PDP or PAE. Moreover, all groups of animals gained weight to a comparable degree during the four week treatment period (S1 Fig). Following completion of four weeks treatment, animals were sacrificed and necropsy performed. Lungs, spleen and tracheobronchial lymph nodes were removed and homogenized. Organ homogenates were plated on agar and three weeks later bacterial burden in these organs was determined by counting the number of Colony Forming Units (CFU). The data are displayed as log_10_ CFU per gram of tissue (Fig 2).

#### Lung burden

When the mean CFU in the lungs of animals treated with R alone was compared to any of the treatments tested in combination with R (R+PAE, R+PZA, R+PDP) there were no statistically significant differences. However, there was a modest trend toward fewer lung CFU in the R+PDP and R+PZA groups compared to other groups. In fact, compared to the untreated group, a statistically significant reduction in CFU was observed for the R+PDP and R+PZA groups, which was not true for the R group or the R+PAE group (Fig 2A).

#### Spleen burden

In spleens, there was again no statistically significant differences in CFU between animals treated with R alone to R+PAE, R+PZA, R+PDP groups. However, when compared to untreated animals, the R+PAE, R+PDP and R+PZA groups all exhibited significantly reduced CFU versus untreated animals, which was not the case for animals treated with R alone (Fig 2B). Moreover, in the three adjunctive treatment groups, there were multiple animals with CFU below the level of detection (10 CFU) in spleens, and the R+PDP group exhibited the most pronounced effect with five of six animals having undetectable CFU. Because *M*. *tuberculosis* bacilli have reached the spleen and the number of spleen CFU have plateaued by four weeks of infection (*i*.*e*. the time of treatment initiation) [54–57] effects on spleen CFU suggest the ability of inhaled PDP and PAE to affect *M*. *tuberculosis* at locations distal to the site of drug delivery in the lung.

#### Lymph Node burden

The CFU burden in tracheobronchial lymph nodes was also evaluated (Fig 2C). For this organ, all treatment groups including the R group exhibited a significant reduction in bacterial burden in comparison to untreated animals and there were no significant differences between groups.

### PDP effects on pathology and granulomas in *Mtb* infected guinea pigs

**Fig 3:**
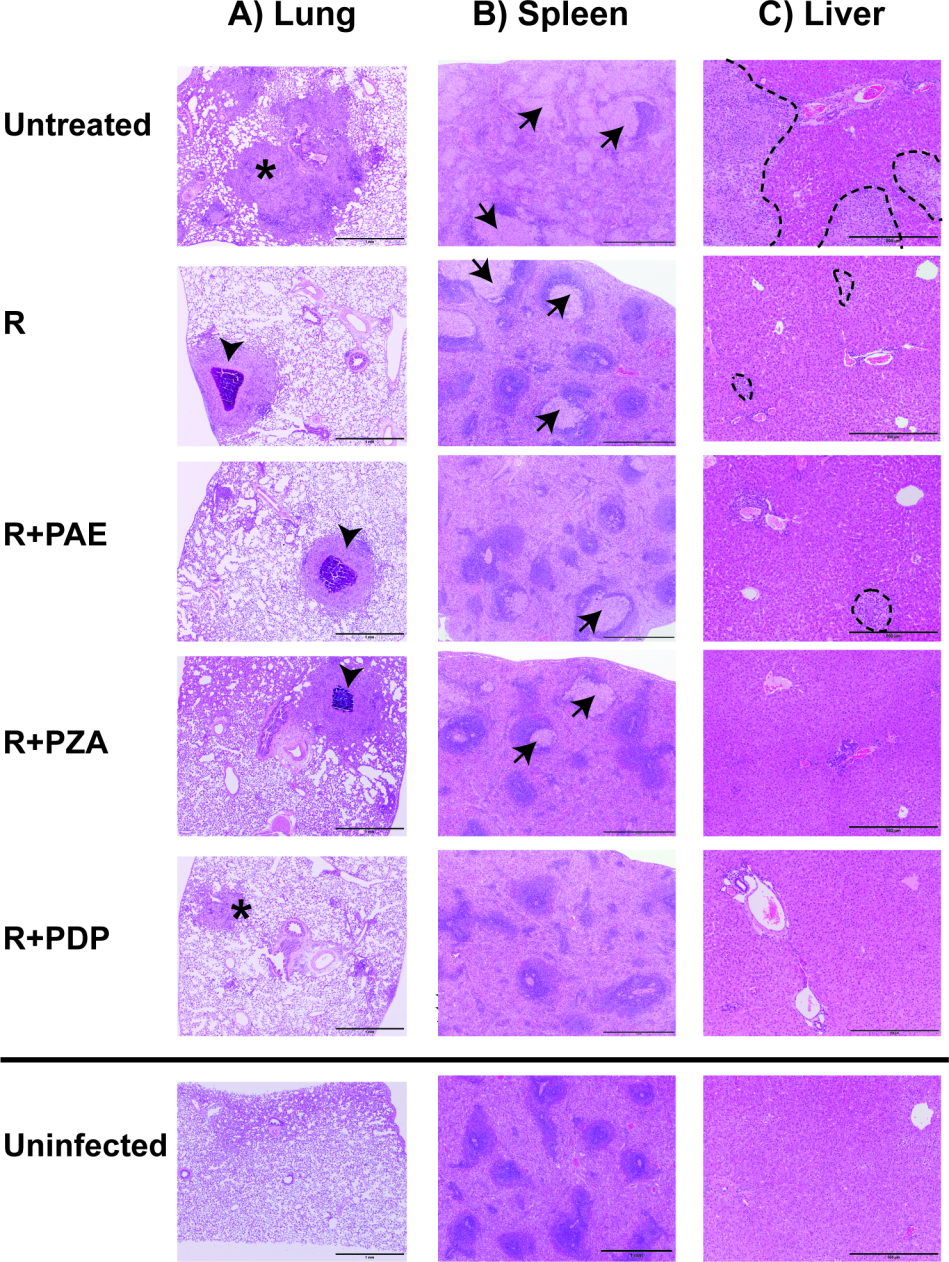
**Images of A) lung, B) spleen, and C) liver histopathologic lesions.** Animals were infected with *M*. *tuberculosis* for four weeks and treated for an additional four weeks with treatment as noted. At eight weeks post-infection, guinea pigs were sacrificed, tissues collected, sectioned and H&E stained. Lungs contained randomly distributed, variably progressed granulomas. While all treatment groups had necrotic granulomas (arrowhead), the untreated group and the R-PDP treatment group had a higher percentage of non-necrotic granulomas (asterisk). The white pulp of spleens is expanded and effaced by granulomatous inflammation (arrows), which appears pale adjacent to the dark basophilic, peripheralized lymphoid follicles. The livers of untreated animals contained large granulomas (outlined) and some livers of R-PAE and RIF (R) treated animals occasionally contained smaller granulomas. Tissues from an age matched representative uninfected guinea pig is included for comparison Bars, 1mm (lung); 1mm (spleen), 500 μm (liver). Images are 40X, 40X and 100X magnification for lung, spleen and liver tissues, respectively.

Extensive histopathologic assessment of disease and appearance of granulomas was performed on haemotoxylin and eosin (H&E) stained tissues from all six animals in each of the groups described above (Fig 3). Tissues from uninfected animals were included as controls.

#### Lung pathology

The lung parenchyma of all groups of *M*. *tuberculosis* infected animals (+/- treatment) contained randomly distributed granulomas. Pulmonary granulomas were comprised of epithelioid macrophages with associated fibrin, surrounding lymphocytes, and rare heterophilic neutrophils (heterophils). Some, more progressed granulomas contained a necrotic center of abundant eosinophilic cellular debris, with some necrotic cores displaying abundant central mineralization (Fig 3A).

As a measure of granuloma involvement and disease, we quantified the percent of lung tissue effaced by granulomas in each of the six animals per group (Table 1).

In untreated animals after eight weeks of infection, 23.4% of lung tissue was affected by granulomas. The R group had 14.4% of lung tissue affected by granulomas and the R+PAE, R+PZA and R+PDP groups had 7.5%, 7.3% and 7.1% affected, respectively. While the difference in percent of lung tissue affected was not statistically significant for any group compared to R, all therapies reduced the level of granuloma involvement with respect to the untreated group. In addition, the combination therapies were more effective than treatment with R alone. When compared to the untreated group, the percent association with granulomas was significantly less (p<0.01) in R+PAE, R+PZA or R+PDP groups, but this was not the case for the R group.

We also measured the amount of necrotic lung tissue across the groups. Although the percent of lung tissue that was necrotic was small for all groups (<1.0%), the R+PDP treated animals exhibited the most striking reduction in the amount of necrosis (Table 2).

The R+PDP group exhibited the least necrosis (0.01%) of all groups and, when compared to R treatment (0.68%), R+PDP was the only group that exhibited significantly less necrosis (p<0.01).

**Fig 4:**
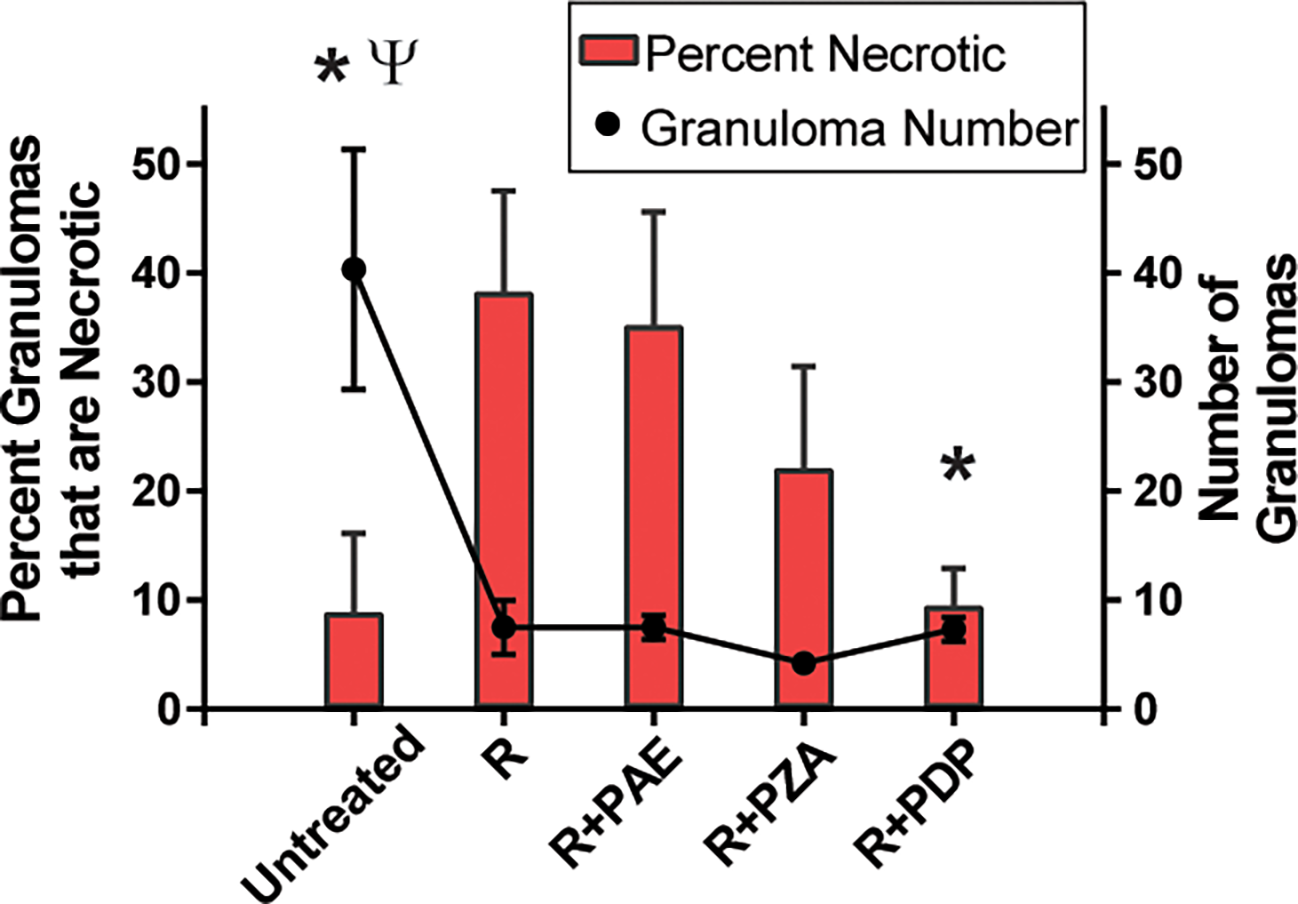
Percent of granulomas that were necrotic. Percent of granulomas that were necrotic in H&E stained lung sections is plotted as red bars. Data is mean percent of six animals. Error bars represent standard error of the mean. *p<0.05 compared to R treated animals using Student’s t-test (p = 0.01 for R+PDP compared to R; p = 0.02 for untreated compared to R). Overlaid on the bar graph is mean number of granulomas counted for animals in each treatment group plotted as black circles. Data is mean of six animals. Error bars represent standard error of the mean. ^ψ^p<0.05 compared to the R group using Student’s t-test (p = 0.02 for untreated compared to R). Raw data counts for necrotic and total granulomas for each of six animals per group is provided in S2 Table.

Given the effect of R+PDP on the amount of necrosis in the lung, we also evaluated individual granulomas and determined if they were necrotic or non-necrotic. This data was reported as percent of granulomas that were necrotic. The percent of granulomas that were necrotic in the R+PDP group (9.5%) was lower than that of all the other treatment groups (38.3% in R, 35.2% in R+PAE and 22.1% in R+PZA) (red bars Fig 4, S2 Table).

Amazingly, this lower percent of granulomas that were necrotic in the R+PDP group was similar to that of the untreated group even though the total number of granulomas in the lungs of the R+PDP group (black circles Fig 4, S2 Table) was reduced from that of untreated animals. The relatively low percentage of necrotic (8.9%) versus non-necrotic lesions in untreated animals was expected as central necrosis is a hallmark of primary granulomas that form from the initial low-dose aerosol infection of the lung. In contrast, the more abundant secondary lesions that form from hematogenous reseeding of bacilli to the lung later in infection are non-necrotic [62]. Compared to the R group, a significantly lower percent of granulomas were necrotic in the R+PDP and untreated groups, which was not the case for R+PAE or R+PZA groups. Thus, it appears that R+PDP affected necrotic (primary) and non-necrotic (secondary) lesions with equal efficacy, such that the necrotic lesions remaining after treatment were proportionally the same as observed in the untreated group. In contrast, the other treatments preferentially resolved non-necrotic lesions resulting in a higher percentage of the remaining granulomas being necrotic. Together, it appears that R+PDP had a distinctive effect on necrotic granulomas both in terms of reducing the overall amount of necrotic lung tissue (Table 2) and in reducing the percentage of remaining granulomas that were necrotic (Fig 4).

#### Spleen pathology

The spleens of all groups of *M*. *tuberculosis* infected animals (+/- treatments) contained splenic granulomas disrupting lymphoid follicles (white pulp). However, the histopathologic changes varied across groups (Table 3, Fig 3B). Factors taken into account in determining degree of pathology were granulomatous lesion distribution, size and frequency as well as tissue architecture changes, types of macrophages present and lymphocyte arrangement (S1 Table).

Following eight weeks of infection with *M*. *tuberculosis*, untreated animals exhibited severe spleen pathology distinguished by the white pulp of the spleens being effaced by large aggregates of macrophages centered on lymphoid follicles (Fig 3B). Macrophages accumulating in the white pulp had abundant, pale cytoplasm (epithelioid macrophages) and rarely exhibited multinucleation. The surrounding red pulp contained increased abundance of circulating heterophils and macrophages. In contrast to untreated animals, the spleens of R+PDP animals had minimal pathology. While the red pulp of R+PDP treated animals displayed increased numbers of circulating heterophils and macrophages, there were only occasional small aggregates of macrophages. Moreover, the white pulp of R+PDP treated animals largely retained normal lymphoid organization. In comparison to the minimal spleen pathology in the R+PDP group, moderate spleen pathology for R, R+PAE and R+PZA groups was observed with increased circulating heterophils and macrophages and varying numbers of granulomas focused on lymphoid follicles.

We additionally quantified the percent of spleen tissue effaced by granulomas in each of the six animals per group (Table 4).

Compared to untreated animals, all treatment groups significantly reduced the percent of spleen affected by granulomas (p<0.01). However, of all the treatment groups, the R+PDP group exhibited the lowest percent of spleen tissue affected (0.1%) although it was not a statistically significant reduction. This quantitation reinforced the histopathologic assessment of the spleen (Table 3).

#### Liver pathology

To determine if effects outside of the lung extended beyond the spleen, we performed histopathologic analysis of livers of the different groups of *M*. *tuberculosis*-infected animals (+/- treatment) (Table 3 and Fig 3C). The livers of untreated animals at eight weeks post-infection exhibited severe pathology that was dominated by randomly scattered, coalescing granulomas comprised of aggregates of epitheliod macrophages and fewer surrounding lymphocytes. Hepatocytes adjacent to these granulomas often displayed degeneration and necrosis. Compared to this severe pathology, liver sections of R+PDP and R+PZA groups had no hepatic granulomas. The R and R+PAE animals displayed mild liver pathology with small granulomas. The livers of all groups also revealed equivalent degrees of non-specific, lymphocytic periportal inflammation, which is consistent with infection.

#### Lymph node pathology

Severe histomorphologic changes were observed for the tracheobronchial lymph nodes of all infected groups (+/- treatment) (Table 3). Tracheobronchial lymph nodes were expanded 10-100X that of uninfected animals, and the normal lymph node architecture was completely effaced by abundant macrophages with fewer admixed lymphocytes, plasma cells, and heterophils. Some graunulomatous lesions in the lymph nodes displayed central necrosis and mineralization.

## Discussion

Due to the distinctive activity profile of PZA and the rise in PZA resistant *M*. *tuberculosis*, there is interest in developing PZA analogs or POA itself to treat TB [44,45,47,50,63]. Here, we tested the efficacy of inhaled PDP, a dry powder combination of POA and PAE, to treat TB. We evaluated PDP as an adjunctive therapy, rather than as monotherapy, since PZA is currently used in combination with other drugs (*e*.*g*. R) and it is widely accepted that any new therapy for TB will be incorporated into multi-drug regimens. While the effects of R+PDP on the bacterial burden in the lung were not significant when compared to treatment with R alone, a striking effect was observed on lung pathology. In comparison to other treatments that preferentially resolved non-necrotic granulomas, R+PDP was better able to reduce necrotic granulomas such that a lower percent of the lung was affected by necrosis and a lower percent of granulomas remaining in R+PDP treated animals were necrotic. Another striking effect of R+PDP was its impact on disease in extrapulmonary organs indicating the ability of inhaled PDP to elicit systemic effects.

### Local (lung) effects of low-dose inhaled PDP

When we examined the bacterial burden in the lung for R+PDP animals we did not observe a statistically significant difference in CFU compared to animals treated with R alone. However, there was a trend, albeit modest, for the R+PDP and the R+PZA groups having greater effects on lung CFU than R treated animals (Fig 2A). Although there was no superior activity on CFU burden for R+PDP versus R+PZA, PDP should have the advantage compared to PZA, in that the POA it delivers (directly or released by hydrolysis of PAE) should be able to act on PZA-resistant as well as sensitive *M*. *tuberculosis* [45,48,50]. However, future studies are warranted to confirm the ability of R+PDP to be effective on PZA resistant disease *in vivo*. 300mg/Kg oral PZA is the guinea pig dose that equates to PZA dose for humans [32]. However, since a dose this high is not feasible for inhaled delivery we used an oral PZA dose of 25mg/Kg for comparison, which accounts for why R+PZA treatment did not exhibit greater efficacy.

More striking than the effect on lung CFU was the effect of R+PDP on lung pathology (Table 1). After 8 weeks infection, 23.4% of lung tissue was affected by granulomas. In comparison, 14.4% tissue was affected by granulomas in the R treated animals and 7.1% tissue was affected by granulomas in the R+PDP group. Pulmonary granulomas are known to form in *M*. *tuberculosis* infected guinea pigs by four weeks of infection [53–56]. Further, in our unpublished studies where guinea pigs were infected in an identical manner by low dose aerosol of *M*. *tuberculosis*, we observed 20.2 +/- 4.3% or 20.1 +/- 3.2% of lung tissue affected by granulomas after four weeks of infection. Thus, it is likely that a significant level of pulmonary granulomas were present at the start of treatment (*i*.*e*. four weeks infection) and that R+PDP treatment resolved pre-existing granulomas in order to achieve 7.1% of lung tissue affected by granulomas. However, we cannot rule out the possibility of treatment also preventing the formation of new granulomas.

### R+PDP was better at resolving necrotic granulomatous inflammation compared to other treatments

Compared to untreated animals, the percent tissue affected by granulomas (Table 1) and the number of granulomas (S2 Table) were significantly reduced in all combination treatment groups, including the R+PDP group. However, in evaluating the percent of lung tissue affected by necrosis, the R+PDP group exhibited the most dramatic reduction of all the groups. Further, among the total number of granulomas in the R+PDP group, the percent of necrotic lesions was similar to that of untreated animals, which had a larger total number of granulomas. This result suggests that the effect of R+PDP in reducing the abundance of granulomas was equally effective in resolving both types of granulomas such that the remaining lung granulomas in the R+PDP group were comprised of the same percentage of necrotic lesions as in untreated animals. This was not the case, however, for the other treatments. While treatment with R, R+PAE or R+PZA also reduced granuloma abundance, these treatments resulted in a significantly higher percent of the remaining granulomas being necrotic. This higher proportion of necrotic lesions remaining with the other treatments is consistent with preferential effects of these treatments on non-necrotic versus necrotic lesions. Thus, compared to the other treatments, R+PDP was distinguished by its better ability to reduce necrotic granulomas.

The effect of PDP on different types of granulomas has potential significance. In low-dose infection of guinea pigs, as performed in this study, the initial inhaled bacilli give rise to primary granulomatous lesions with necrosis while subsequent hematogenous reseeding of the lungs (~3 weeks post-infection) leads to secondary granulomatous lesions that do not undergo necrosis. The T cell response established during infection is the likely explanation for the limited progression of the secondary lesions (sometimes termed post-primary lesions) [62]. As reported previously [64–66] and as we observed for R, R+PAE or R+PZA groups, TB treatments preferentially clear non-necrotic (secondary) lesions over primary necrotic lesions. In one such study, combination R+isoniazid+PZA therapy had this effect of preferentially resolving non-necrotic lesions [64]. Moreover, a study of standard multi-drug therapy on bacilli in excised primary and secondary lesions from guinea pigs reveals faster sterilization of secondary lesions over primary lesions [54]. Thus, our finding that R+PDP is better equipped in resolving necrotic granulomatous inflammation, compared to other therapies, suggests a different mechanism of clearing granulomas for R+PDP. It is worth noting that current methodologies do not allow us determine if the non-necrotic granulomas remaining in animals treated with inhaled R+PDP are primary granulomas that have resolved the necrotic center or if they are secondary granulomas that failed to progress to necrosis. Given that R+PDP and R+PZA elicited comparable modest effects on the bacterial burden in the lung (Fig 2A), it is interesting to discover that the two treatments affected necrotic granulomas to a different degree (Table2, Fig 4). Thus, the importance of the effects of R+PDP may not be best measured by reduced lung CFU but, rather, by the better ability of R+PDP to target bacteria that are persisting in the distinctive environment of a necrotic granuloma. Our results reveal the exciting possibility for developing therapies that improve clearance of the more recalcitrant primary necrotic granulomas as a strategy to prevent disease relapse when treatment ends or to treat latent *M*. *tuberculosis* harbored in necrotic granulomas. Future studies are warranted to evaluate these possibilities including the potential of using PDP as an add-on to more aggressive combination chemotherapy as an approach to achieve more complete sterilization.

### Systemic effects of low-dose inhaled PDP

When compared to animals treated with R alone, R+PDP treatment did not significantly reduce the bacterial burden in the spleen. However, there was a trend toward fewer spleen CFU in the R+PDP group compared to R. In fact, five of six animals in the R+PDP had undetectable CFU while only one of six animals had undetectable CFU in the R group. R+PDP was also associated with reduced histopathology in spleen and livers (Fig 3, Tables 3 and 4). Our observation of PDP effects distal to the site of pulmonary delivery reinforces our prior observation of nebulized PAE having effects on CFU in the spleen [15]. While it is possible that the spleen effects are due to drug in the lung limiting *M*. *tuberculosis* dissemination to the spleen, this explanation seems unlikely to account for the results. By the time drug treatment was initiated at four weeks post-infection, *M*. *tuberculosis* has reached the spleen, the number of spleen CFU has plateaued and splenic granulomas have formed [54–57]. Consequently, the effects observed in the spleen likely reflect an ability of R+inhaled PDP or PAE to clear pre-existing bacteria and pathology.

Future PK studies will be required to directly determine the dose and distribution of inhaled PDP. However, the calculated doses for inhaled PDP or PAE (potential deliverable POA doses of up to 1.4mg/Kg or 4.3mg/Kg of POA, respectively) were relatively low and the dose reaching the systemic circulation is expected to be even lower. Thus, the results in the spleen are particularly striking in light of the low doses of inhaled drug used. An intriguing possibility is that the extrapulomonary effects observed with low circulating levels of PDP or PAE reflect host directed effects as opposed to direct anti-bacterial effects. The potential for PZA or PZA analogs having immunomodulatory effects is suggested previously [67–69]. Because our study tested inhaled low-levels of PDP and PAE in combination with R, it may be that such host directed effects improve the efficacy of the orally delivered R.

### Differences in the performance of PDP and PAE

This study also revealed a trend in inhaled PDP (POA+PAE combination) being more effective than inhaled nebulized PAE. Most notably, the effects on necrotic granulomas differed between R+PDP and R+PAE groups. We were surprised to see such differences for several reasons. First, less PAE was delivered by inhaled PDP compared to the nebulized PAE solution. Second, we had not expected the POA in the PDP formulation to be particularly effective since, at least when delivered orally as monotherapy to mice, POA at tolerable doses shows minimal efficacy [48]. Third, in estimating the potential POA doses delivered/released by PDP or the PAE solution, a minimum of three-fold less POA was delivered by PDP versus PAE (S1 File). The most likely explanation for the greater effects of inhaled PDP is that the POA it directly delivered to the lungs was active and more effective than inhaled PAE and the POA it could release. Direct administration of POA to the lungs was proposed previously [44,48]. Our results warrant future studies of inhaled dry powders of POA alone. Importantly, there were no signs of damage to the lungs or tracheas of animals treated with inhaled PDP or PAE, beyond that associated with disease.

### Conclusion

Motivated by a desire to develop an inhaled PAE dry powder to treat TB, the studies presented here revealed inhaled PDP, a dry powder combination of POA and PAE, as working at low levels compared to standard oral therapies, having local and systemic effects and being distinguished by a better ability to resolve necrotic granulomas than other therapies. Several criteria suggest that inhaled PDP is more effective than inhaled PAE, which raises the possibility of direct delivery of POA to the lungs being effective. Combined with the fact that, at least *in vitro*, POA and PAE work on the largest category of PZA-resistant *M*. *tuberculosis* organisms, the results presented here argue for further development of inhaled PDP or POA alone as an adjunctive therapy for treating TB.

## Supporting information

S1 File Dose calculations. (DOCX)

S1 Fig Mean body weight of guinea pig groups at the start and end of the four week drug treatment period. * p<0.01 comparing starting and ending mean weight for groups of animals using Student’s t-test. Error bars represent standard deviation of the mean.
(TIF)

S1 Table Descriptive terms and parameters used to score histopathology in extrapulmonary organs. (DOCX)

S2 Table Granulomas counted across groups of six guinea pigs. (XLSX)
