# Correction: Efficacy of pyrazinoic acid dry powder aerosols in resolving necrotic and non-necrotic granulomas in a guinea pig model of tuberculosis

**DOI:** 10.1371/journal.pone.0207257

**Published:** 2018-11-05

**Authors:** The PLOS ONE Staff

As a result of the typesetting process, Tables 1–4 have been incorrectly placed between the “Statistical methods.” sub-section of the Materials and methods section and the beginning of the Results section in the online version of the article. In the PDF version of the article, Tables 1–4 have been incorrectly placed at the beginning of the Results section. The publisher apologizes for the error.

Table 1 should appear between the second and third paragraphs of the “Lung pathology.” sub-section of the Results section.

Table 2 should appear between the fourth and fifth paragraphs of the “Lung pathology.” sub-section of the Results section.

Table 3 should appear between the first and second paragraphs of the “Spleen Pathology.” sub-section of the Results section.

Table 4 should appear between the third and fourth paragraphs of the “Spleen Pathology.” sub-section of the Results section.
