# Supplementary material for: Efficacy of pyrazinoic acid dry powder aerosols in resolving necrotic and non-necrotic granulomas in a guinea pig model of tuberculosis
Source: PLoS One. 2018 Sep 27;13(9):e0204495. doi: 10.1371/journal.pone.0204495 (PMC6160074; doi:10.1371/journal.pone.0204495)
Supplement: S1 File — (DOCX) [file pone.0204495.s001.docx]

**S1 File**

| **Estimated Doses*** | | | | | |
| --- | --- | --- | --- | --- | --- |
|  | PAE  (mg/Kg) | POA from ester (mg/Kg) | POA alone (mg/Kg) | Total POA (mg/Kg) | Total Powder  (mg/Kg) |
| Nebulized PAE | 5.8 | 4.3 | NA | 4.3 | NA |
| Inhaled PDP | 0.3 | 0.2 | 1.2 | 1.4 | 3.9 |

***** Due to biological variation in physiology and pulmonary function dose ranges around the calculated values would be anticipated experimentally.

***Dose Calculation*** [[1](#_ENREF_1)]

Drug [mg] x duration [min] x *breathing [breaths.min^-1^] x ^+^tidal [L.breath^-1^] x Lung [%]

Mass of exposure frequency volume Fraction

Exposure chamber volume [L] x Turnover Number

***Definitions***

Drug mass –

- Nebulized solution - calculated from the volume of droplets delivered, the concentration of the drug and assuming unit density (g/mL) of the solution.
- Dry powder: calculated from the known mass of drug (both PAE and POA) in the PDP delivered.

Guinea pig breathing frequency is 90 breaths/min and tidal volume is 1.8 mL

(1.8 x 10^-3^ L) [[2](#_ENREF_2)]

Lung fraction is 6% based on the MMADs for all aerosol being in the range 2.5-3.0μm and GSDs of 1.6-1.7 [[3](#_ENREF_3)].

Exposure time 20 min

Exposure chamber volume 2.5L/min

Turnover Number: Aerosol was delivered 5 times in 20 minutes based on decay to undetectable limits after each bolus. Consequently, the total volume in which the dose was distributed was 5 times the exposure chamber volume.

***Dose from Nebulized Solution***

1mL of a 15% solution of PAE (density 1g/mL) was administered over 20 minutes.

150mg x [20min x 90 breaths/min x 1.8 x 10-3 L/breath x 0.06]

[2.5 x 5 L]

= 150 x 15.55 x 10^-3^ = 2.33mg deposited.

Animals were 400g at time of dosing so this corresponds with

**A nebulized PAE dose of 5.825 mg/Kg**

([1000/400] x 5.825)

PAE (MW 167.1) consists of POA (MW 124.1 Da) and the propyl ester (43 Da).

Therefore, PAE contains a fraction of 0.743 POA.

The Nebulized **PAE dose of POA = 4.33 mg/Kg** (5.825 x 0.743)

***Dose from Dry Powder (PDP)***

100mg of PDP was delivered containing 8.5% PAE [8.5mg] and 30.5% POA [30.5mg] was administered over 20 mins.

PAE DOSE

8.5 x 15.55 x 10^-3^ = 0.132mg.

Animals were 400g at time of dosing so this corresponds with a

**Dry Powder PAE dose of 0.33 mg/Kg** ([1000/400] x 0.132).

Considering the proportion of POA (0.743) in PAE the dry powder then

**Dry powder PAE dose of POA is 0.245mg**

POA DOSE

30.5 x 15.55 x 10^-3^ = 0.474mg

Animals were 400g at time of dosing so this corresponds with a

**Dry powder POA dose of 1.186mg/Kg** ([1000]/400] x 0.474)

TOTAL POA DOSE FROM PDP

The total POA dose (mg/Kg, POA_Total_) from PDP is the sum of the POA alone (1.188mg/Kg) and POA from PAE (0.245 mg/Kg).

**POA_Total_ = 1.186 + 0.245 = 1.431mg/Kg**

TOTAL DOSE OF POWDER FROM PDP

The total dose of powder inhaled includes the additives that make up the rest of the spray dried particle. 39% of the particle consists of POA alone and PAE. The remainder is maltodextrin and leucine.

PDP_Total_ = ([1.186 + 0.33] x [100/39]) = 3.89 mg/Kg

**References**

1. Suarez S, Kazantseva M, Bhat M, Costa D, Hickey AJ. The influence of suspension nebulization or instillation on particle uptake by guinea pig alveolar macrophages. Inhalation toxicology. 2001;13(9):773-88.

2. Chaffee VW. Surgery of laboratory animals. In: Melby EC, Akltman NH, editors. Handbook of Laboratory Animals. Volume 1. Boca Raton, FL: CRC Press; 1974. p. 233-73.

3. Schreider JP, Hutchens JO. Particle deposition in the guinea pig respiratory tract. Journal of Aerosol Science. 1979;10:599-607.
