# Supplementary figures and images for: Efficacy of pyrazinoic acid dry powder aerosols in resolving necrotic and non-necrotic granulomas in a guinea pig model of tuberculosis
Source: PLoS One. 2018 Sep 27;13(9):e0204495. doi: 10.1371/journal.pone.0204495 (PMC6160074; doi:10.1371/journal.pone.0204495)

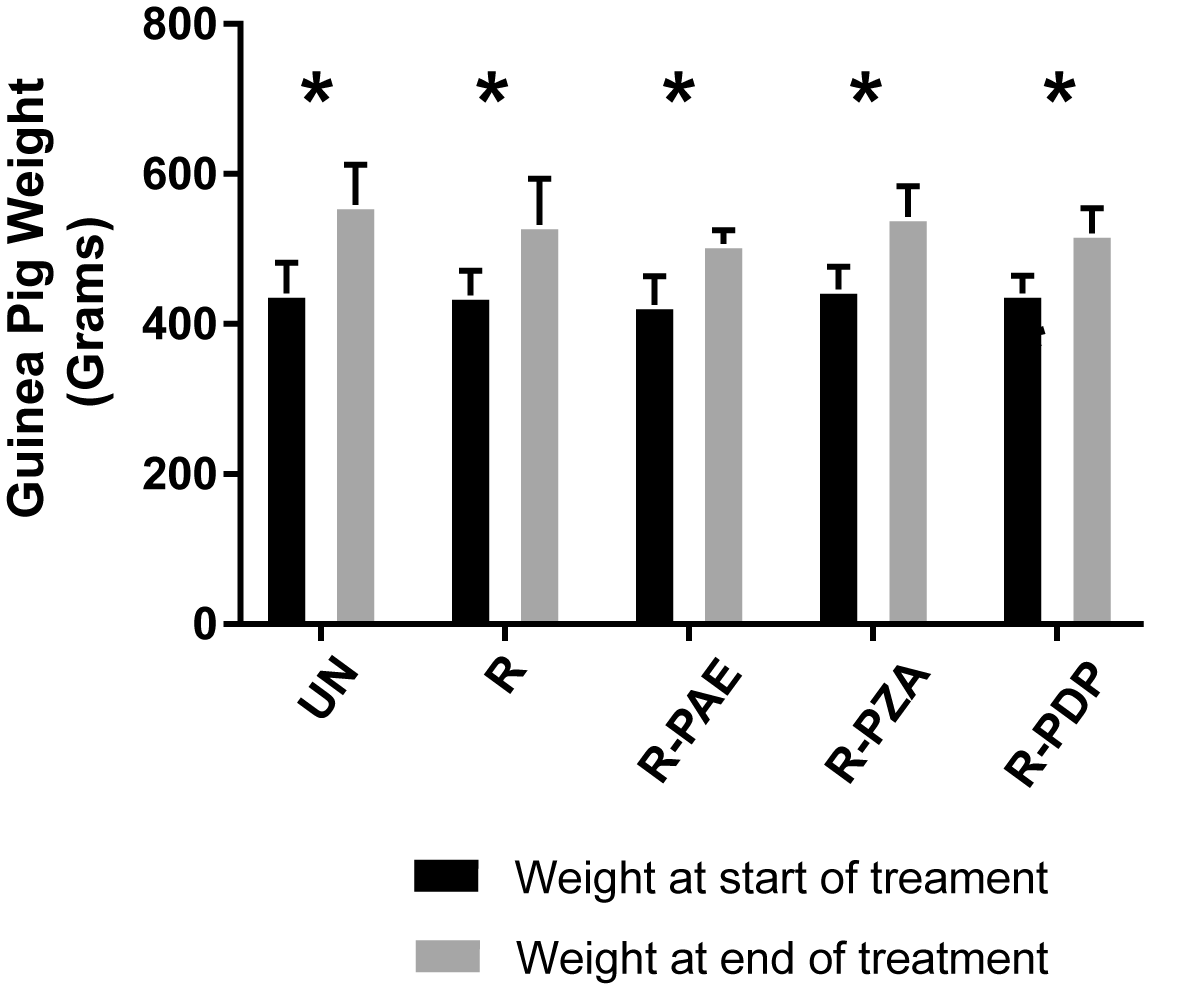

Supplement: S1 Fig — * p<0.01 comparing starting and ending mean weight for groups of animals using Student’s t-test. Error bars represent standard deviation of the mean. (TIF) [file pone.0204495.s002.tif]
