# Supplementary material for: Efficacy of pyrazinoic acid dry powder aerosols in resolving necrotic and non-necrotic granulomas in a guinea pig model of tuberculosis
Source: PLoS One. 2018 Sep 27;13(9):e0204495. doi: 10.1371/journal.pone.0204495 (PMC6160074; doi:10.1371/journal.pone.0204495)
Supplement: S1 Table — (DOCX) [file pone.0204495.s003.docx]

**S1 Table. Descriptive terms and pathology used to score histopathology in extrapulmonary organs**

| **Score** | **Granulomatous lesion distribution** | **Size** | **Frequency** | **Tissue Architecture**  **Changes** | **Macrophage Types** | **Lymphocyte Arrangement** |
| --- | --- | --- | --- | --- | --- | --- |
| Minimal | Multifocal, discrete | Small | Rare | Minimal | Histiocytes, epitheliod | Rare, scattered cells |
| Mild | Multifocal, discrete to occasional coalescence | Small and few medium | Infrequent | Slightly expanding tissue | Histiocytes, epitheliod | Rare, scattered cells to very small aggregates |
| Moderate | Multifocal, coalescing | Medium | Common | Expanding tissue, some disruption of normal structure | Histiocytes, epitheliod and foamy | Small aggregates, typically at margins |
| Severe | Multifocal, coalescing | Large | Very frequent | Effacing normal structure | Histiocytes, epitheliod and foamy | Aggregates at margins or within granulomas |
